# Early onset of Chanarin-Dorfman syndrome with severe liver involvement in a patient with a complex rearrangement of ABHD5 promoter

**DOI:** 10.1186/1471-2350-15-32

**Published:** 2014-03-14

**Authors:** Sara Missaglia, Eugenia Ribeiro Valadares, Laura Moro, Eleonora Druve Tavares Faguntes, Raquel quintão Roque, Bruno Giardina, Daniela Tavian

**Affiliations:** 1 CRIBENS-Laboratory of Cellular Biochemistry and Molecular Biology, Catholic University of the Sacred Heart, Milan, Italy; 2 Psychology Department, Catholic University of the Sacred Heart, Milan, Italy; 3 Department of Propedêutica Complementar, Universidade Federal de Minas Gerais, Belo Horizonte, Brazil; 4 Department of Pharmaceutical Sciences, University of Piemonte Orientale “A. Avogadro”, Novara, Italy; 5 Department of Pediatrics, Hospital das Clínicas, Universidade Federal de Minas Gerais, Belo Horizonte, Brazil; 6 Ambulatório de Erros Inatos do Metabolismo, Hospital das Clínicas, Universidade Federal de Minas Gerais, Belo Horizonte, Brazil; 7 Laboratory of Clinical Molecular Diagnostics, Institute of Biochemistry and Clinical Biochemistry, Catholic University, School of Medicine, Rome, Italy

**Keywords:** Chanarin-Dorfman syndrome, ABHD5, Lipid disorder, Liver involvement, Ichthyosis

## Abstract

**Background:**

α/β-hydrolase domain-containing protein 5 (ABHD5) plays an important role in the triacylglycerols (TAG) hydrolysis. Indeed, ABHD5 is the co-activator of adipose triglyceride lipase (ATGL), that catalyses the initial step of TAG hydrolysis. Mutations in ABHD5 gene are associated with the onset of Chanarin-Dorfman syndrome (CDS), a rare autosomal recessive lipid storage disorder, characterized by non-bullous congenital ichthyosiform erythroderma (NCIE), hepatomegaly and liver steatosis.

**Case presentation:**

We describe here a 5-years-old Brazilian child who presented with NCIE at birth and diffuse micro and macro-vesicular steatosis on liver biopsy since she was 2 years old. Molecular analysis of coding sequence and putative 5′ regulatory region of ABHD5 gene was performed. A homozygous novel deletion, affecting the promoter region and the exon 1, was identified, confirming the suspected diagnosis of CDS for this patient. RT-PCR analysis showed that the genomic rearrangement completely abolished the ABHD5 gene expression in the patient, while only a partial loss of expression was detected in her parents. This is the first report describing the identification of a large deletion encompassing the promoter region of ABHD5 gene. The total loss of ABHD5 expression may explain the early onset of CDS and the severe liver involvement. After molecular diagnosis, the patient started a special diet, poor in fatty acids with medium chain triglycerides (MCT), and showed hepatic and dermatologic improvement in spite of severe molecular defect.

**Conclusions:**

This case report extends the spectrum of disease-causing ABHD5 mutations in CDS providing evidence for a novel pathogenic mechanism for this rare disorder. Moreover, our preliminary data show that early diagnosis and prompt treatment of neutral lipid accumulation might be useful for CD patients.

## Background

Neutral lipid storage diseases (NLSDs) are a heterogeneous group of lipid metabolic disorders, characterized by a deficit in the degradation of the triacylglycerol and its abnormal accumulation in cytoplasmic lipid droplets (LDs) present in most tissues, including skin, liver and peripheral blood. These rare autosomal recessive syndromes comprise the Chanarin-Dorfman syndrome (CDS; MIM 275630), or Neutral Lipid Storage Disease with Ichthyosis (NLSDI) [1], and the Neutral Lipid Storage Disease with Myopathy (NLSDM; MIM 610717) [2,3]. The clinical symptoms of CDS, described by Dorfman [4] in 1974 and by Chanarin [5] in 1975, are characterized by non-bullous congenital ichthyosiform erythroderma (NCIE) [6], hepatomegaly and liver steatosis [7]. Muscle and hepatic enzymes levels are often elevated. Other variable features are muscle weakness, ataxia, neurosensory hearing loss, sub-capsular cataracts, nystagmus, strabismus and mental retardation [8]. Patients are sometimes born as collodion babies [9,10]. The clinical diagnosis is based on observation of Jordans’ bodies, characteristic cytoplasmatic vacuoles, in the granulocytes [11].

The onset of CDS is caused by mutations in the ABHD5 gene [1]. This gene is located on chromosome 3p21, contains seven exons and codifies for the α/β-hydrolase domain-containing protein 5, a protein of the esterase, lipase and thioesterase subfamily. The human ABHD5 is a 349 amino acid long protein with a molecular mass of 39 kDa, characterized by a pseudo-catalytic domain and a lipid binding motif. In the pseudo-catalytic domain, the usual serine is replaced by asparagine [12]. Consequently, ABHD5 does not present enzymatic hydrolase activity but co-activates adipose triglycerides lipase (ATGL) [13]. In addition, ABHD5 preforms a lysophosphatidic acid acyltransferase (LPAAT) function [14]. It has been recently demonstrated in mice that inflammatory stimuli increase stress kinase levels in the liver. Subsequently, these cytokines promote generation of phosphatidic acid (PA) from LPAAT activity of ABHD5. PA activates stress kinases, decreasing hepatic insulin signalling [14].

In the present study we describe the clinical and molecular characterization of a CDS patient from Brazil. The genetic analysis of ABHD5 detected a novel deletion in the promoter region, confirming the diagnosis of Chanarin-Dorfman syndrome.

## Case presentation

**Figure 1:**
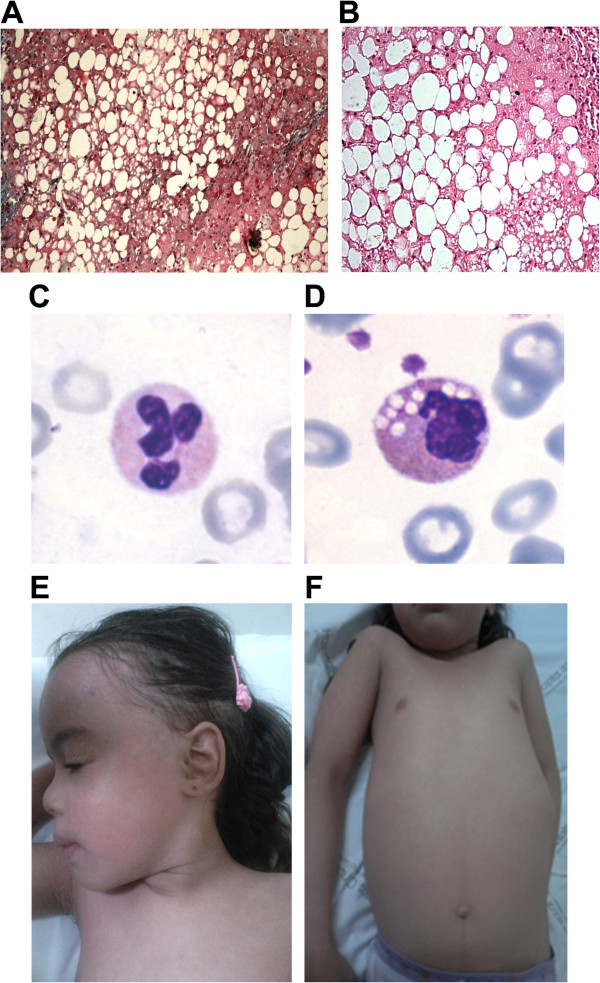
**Clinical phenotype of CDS patient. A** and **B)** Liver biopsy stained with Trichrome- Magnification 100× and with Hematoxylin and Eosin (HE)-Magnification 200×; **C** and **D)** Buffy coats stained with May-Grünland Giemsa (MGG) from control subject and patient: Jordans’ bodies can be detected only in patient’s granulocytes-Magnification 100×; **E** and **F)** Erythema and fine desquamation of the skin on entire body surface.

Our patient is a five-year-old Brazilian girl, second child of non-consanguineous parents. Her perinatal period was normal, but section was performed at 35 weeks of pregnancy. At birth she had a weight of 2.575 g, a length of 45 cm and an occipital frontal circumference of 34 cm. Her skin was diffusely red and scaly, without hair, eyelashes and eyebrows. A diagnosis of congenital ichthyosis was made at that time. Since the early days of life she was followed by dermatologist, who prescribed an emolient (aquaphor) topical cream. When she was 2 years old, in order to start a new dermatological treatment with hydrocortisone, liver function tests were carried out revealing mildly elevated values for aspartate aminotransferase test (AST) and alanine aminotransferase test (ALT), while normal values for gamma glutamyl transpeptidase (GGT), serum albumin and bilirubin. Nevertheless massive hepatomegaly was detected. At the age of 2 years 5 months liver biopsy showed diffuse micro and macro-vesicular steatosis, without fibrosis (Figure 1A and B). At age of 3 years, multiple laboratory tests were performed: AST and ALT were mildly elevated (85 IU/L and 77 IU/L, respectively), GGT was elevated (28 IU/L), total bilirubin (0.2 mg/dL) and serum albumin (4.4 g/dl) were normal, alkaline phosphatase (AP) activity was of 93% and international normalized ratio (INR) was 1.03. At age of 4 years 3 months, Jordans’ bodies were observed in granulocytes (Figure 1C and D), AST (46 IU/L) and serum albumin (3.8 g/dl) were normal, ALT (64 IU/L) was slightly elevated, GGT (35 IU/L) was increased, AP activity was of 72.5% and INR 1.2. Lipid analysis showed a normal level of total cholesterol (112 mg/dL) and triglycerides (73 mg/dL). She did not present clinical evidence of myopathy, but there was a mild elevation of creatine kinase. When she was 4 years 9 months, her liver was palpable 10 cm below the right costal margin and low fat diet with medium chain triglycerides (MCT) was started, maintaining the usual dermatological treatment. Topical ointment was started at birth, at which age the skin lesions improved. At age of 5 years 5 months, she had a weight of 19.1 kg and a length of 109.5 cm, her skin was diffusely fine (Figure 1E and F) and liver was palpable 7.5 cm below the right costal margin.

Since age of 2 years she had recurrent acute otitis media and at age of 4 years mild conductive hearing loss was detected by audiometry and impedanciometry.

### Materials and methods

#### Genomic analysis

Genomic DNA was extracted from blood samples using Gentra Puregene Kit (Qiagen). All ABHD5 exons (GeneBank NG_007090.3) were PCR amplified [9]. Also the sequence spanning 5 kb upstream from the ATG start codon was analysed by long-range PCR using forward primer 5′-GCAGCCAGGCCATTGAAGCC-3′ and reverse primer 5′-CTCAGTGCAACGAGGAAGTT-3′. Amplification was performed in a total volume of 50 μl containing 50 ng of genomic DNA, 10× PCR × Enhancer solution 1× (Invitrogen), 10× PCR × Amplification Buffer, MgSO_4_ 2 mM, dNTP 4 mM (Fermentas Inc), oligonucleotide primers 1 μM and GoTaq® DNA polymerase (1 U; Promega Co). Thermal cycling (PTC-200; MJ Research Inc) consisted of 5 min of initial denaturation at 95°C followed by 36 cycles of 94°C (40 s), 56°C (30 s) and 72°C (1 min) with a final extension step of 5 min at 72°C. All PCR products were purified and sequenced on 3730 DNA Analyzers by the BigDye® Terminator V1.1 Cycle Sequencing Kit (Applied Biosystems). PROSCAN Program Version 1.7 was used to scan the putative promoter region of ABHD5 gene and RepeatMasker software to identify repeat elements near the deleted sequence.

#### Comparative RT-PCR

1 μg RNA isolated from PAXgene blood tubes by PAXgene blood RNA kit (Preanalytix) was converted into cDNA. 50 ng of cDNA were used to perform RT-PCR amplification of ABHD5 mRNA [9]. Integrity of RNA and comparable cDNA synthesis in all samples tested were confirmed using glyceraldehyde 3-phosphate dehydrogenase (GAPDH) gene expression.

### Results

**Figure 2:**
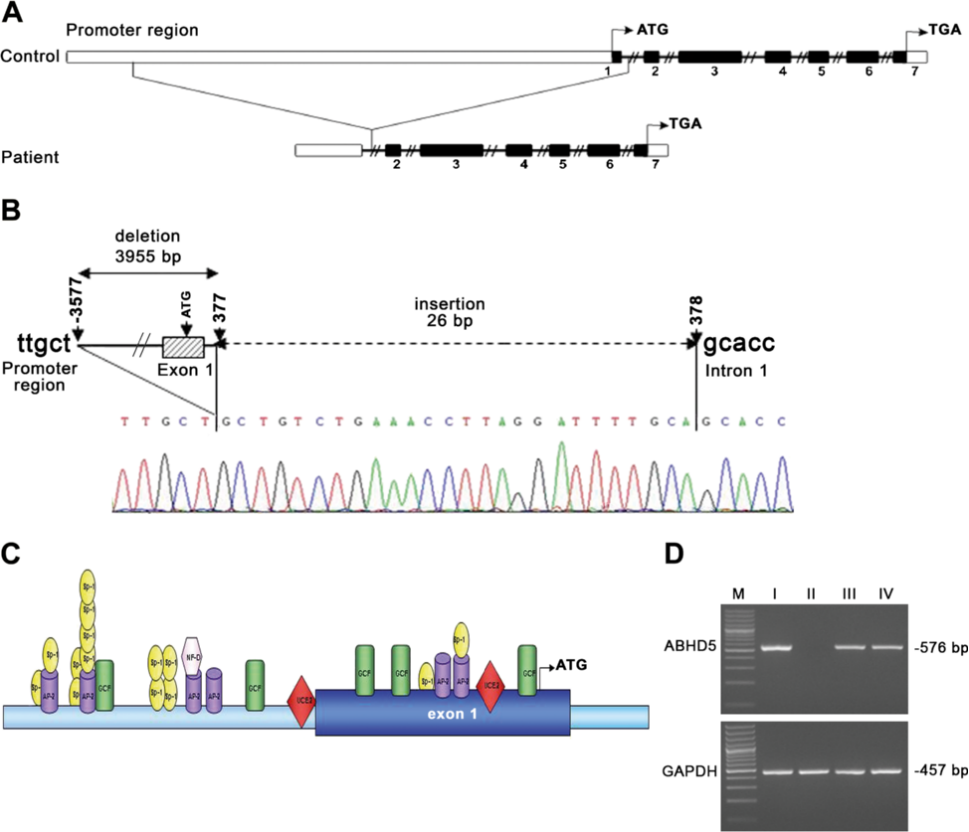
**Identification of ABHD5 large deletion. A)** Diagram of the large deletion identified in Brasilian patient. Normal sequence of ABHD5 and promoter region is aligned with the deleted sequence. **B)** Scheme of rearrangement: deletion removes 3454 bp of the promoter sequence, exon 1 and 331 bp of the intron 1. DNA repair was accompanied by an insertion of 26 bp (GCTGTCTGAAACCTTAGGATTTTGCA), indicated by a dashed line, where the DNA breakpoints rejoined. Electropherogram of ABHD5 gene deletion in CDS patient is shown below the scheme. **C)** Schematic representation of the promoter region identified at 0.3 kb from the ATG start codon with all transcription factors. **D)** ABHD5 expression evaluated by comparative RT-PCR. Picture of gel shows lack of ABHD5 mRNA in the affected proband. M: 100-bp molecular weight marker. Lane I: control. Lane II: CDS patient. Lane III: father. Lane IV: mother.

ABHD5 gene analysis detected a 3955 bp homozygous novel deletion associated with a 26 bp insertion (NG_007090.3: g.43728907_43732862del3955ins26). The rearrangement affected a region located 3.5 kb upstream of the ATG start codon and also the exon 1 (Figure 2A and B). This mutation was submitted to GenBank (accession number KF169942).

Proscan 1.7 software was used to identify the promoter region, from the ATG start codon until 5000 bp upstream. Two putative sequences were identified: the first was localized at 0.3 kb and the second at 5 kb, both comprising transcription factors as AP-2, Sp1, GCF, NF-D and UCE.2 (Figure 2C). The large deletion completely removed the first putative promoter region. A comparative ABHD5 RT-PCR analysis was performed from the peripheral blood of the CDS patient, her parents and a control subject to evaluate whether the mutation could affect gene expression. In the cDNA from patient, no ABHD5 mRNA could be detected. In parents, only a partial decrease of ABHD5 mRNA level was observed in comparison with control cDNA sample (Figure 2D).

In order to verify whether the deletion could be determined by the presence of repeat sequences, the analysis of the region around breakpoints was performed with the RepeatMasker software. High density of Alu and LTR repeats has been identified in the ABHD5 promoter region, although not strictly adjacent to the breakpoint flanking region. Nevertheless, it cannot be excluded that these repeat sequences could be involved in the molecular mechanism causing this complex rearrangement, since it is known that they usually promote recombination events.

### Discussion

Chanarin-Dorfman syndrome is a multisystem genetic disorder involving many tissues. Although the majority of cases come from Mediterranean and Middle East regions, CDS patients were also reported from Japan and India. In this study, we have identified a novel homozygous large deletion in a Brazilian patient, child of non-consanguineous parents. The genomic rearrangement affects the promoter region and the ATG start codon of ABHD5 gene, totally abrogating its expression. Until now, 63 CDS cases have been reported all over the world; molecular diagnosis was performed for 52 of them. Thirty-three different ABHD5 mutations have been described, including deletions, insertions, missense, nonsense and frameshift variations. Even though ABHD5 deletions have been previously reported, the rearrangement, described in this study, is the first localized in the promoter region and it results in the lack of mRNA and protein production. To our best knowledge, 11 different mutations causing truncated proteins have been described in children younger than 6 years (21% of patients for whom molecular diagnosis was done) [8,9,15-18]. These genetic variations should lead to ABHD5 total or partial loss and might explain the early onset of CDS. In particular, two mutations, S17X and S33X, might result in the absence of ABHD5 protein. The first one was identified in a two-year-old patient and it was associated with development of severe liver cirrhosis in the infancy [16]. The second mutation was detected in a five-year-old girl [8]. This patient was characterized by hepatomegaly, elevated levels of CK and diffuse weakness. Similarly, the data reported here suggest that the novel large deletion may be responsible for early onset of the disease and for severe liver involvement, documented by massive fat accumulation observed in our patient. In fact, the biopsy reveals marked steatosis, even though the liver function is normal so far, without hepatic decompensation. The clinical course of all young CDS patients will be important to follow in order to monitor the hepatic damage. The lack of ABHD5 synthesis may result in an excessive and premature accumulation of triacylglycerol in cytoplasmatic lipid droplets, particularly in liver. As a result, the CDS patients are characterized by hepatomegaly, steatosis and/or cirrhosis. In these patients, ATGL is not mutated but its activity is strongly decreased, since its activator is not produced. In addition, the loss of ABHD5 protein also causes the abolition of LPAAT activity and the lack of PA production. Recent studies show that, in mouse liver, PA acts as messenger to promote the activation of stress kinases (IKK-β, S6K1 and mTOR) in response to inflammatory stimuli, such as high-fat diet and lipopolysaccharide treatment. In ABHD5 knockdown mice, the abrogation of PA synthesis results in the decrease of activity of this pathway, causing severe hepatic involvement [14]. It would be important to investigate in human, and in particular in CDS patients, if the lack of PA synthesis increases liver injury.

Whereas there is not specific pharmacologic treatment for CDS, some authors recommended a low fat diet supplemented with MCT to improve the liver findings and a treatment with emollients for ichthyosis. A special diet in the infancy would decrease the liver size and normalize the hepatic enzymes. To date, 10 children, younger than 6 years (17% of patients), were put on a low fat diet and 4 of them showed a decrease of liver size [16,19-21]. A six-year-old girl, CD patient who showed hepatomegaly, started a diet supplemented with MCT and, after 4 months, the liver size was decreased [19]. A reduction of liver fibrosis and a normalization of hepatic enzymes were observed in a two-year-old patient, after treatment with a low fat diet combined with the use of ursodeoxycholic acid (UDCA) and vitamin E [16]. In 1997 Kakourou et al. described liver decreased by 50% in a three-year-old boy after a medium-chain TG diet. The same patient, at age of 8 years and still on the low fat diet, showed a normal liver size [20]. Recently, Mitra et al. reported a three-year-old CDS patient who presented hepatomegaly (10 cm below the costal margin) and mild splenomegaly and who was put on a low fat diet with UDCA and vitamin E. After 1 year, liver size decreased (4 cm below the costal margin) and splenomegaly was not available [21]. Similarly, our patient started using UDCA, vitamin E and a special diet, poor in fatty acids with MCT. 1 year after treatment, the child showed an improvement of liver size (from 10 cm to 7.5 cm below the costal margin) and skin condition.

## Conclusions

For the first time, our study provides evidence that complex genomic rearrangements of ABHD5 promoter may be associated with CDS onset. Therefore, the ABHD5 complete molecular screening, comprising the promoter region, is important in order to validate the clinical diagnosis and, although the role of special diet remains controversial, an early initiation of low fat diet might improve the liver condition. Additional studies are warranted to clarify the benefits of low fat diet in CDS patients. Moreover, will be of great interest to evaluate possible dermatological improvement.

## Consent

Informed consent for genetic analysis was obtained from the study participants. Moreover, as the patient is a child, written informed consent was obtained from her mother for publication of this Case report and any accompanying images. A copy of the written consent is available for review by the Editor of this journal.

## Abbreviations

ABHD5: α/β-hydrolase domain-containing protein 5; TAG: Triacylglicerol; ATGL: Adipose triglyceride lipase; CDS: Chanarin-Dorfman syndrome; NCIE: Non-bullous congenital ichthyosiform erythroderma; MCT: Medium chain triglycerides; LD: Lipid droplet; NLSDI: Neutral lipid storage disease with ichthyosis; NLSDM: Neutral lipid storage disease with myopathy; LPAAT: Lysophosphatidic acid acyltransferase; PA: Phosphatidic acid; AST: Aspartate aminotransferase test; ALT: Alanine aminotransferase test; GGT: Gamma glutamyl transpeptidase; AP: Alkaline phosphatise; INR: International normalized ratio; UDCA: Ursodeoxycholic acid.

## Competing interests

The authors declare that they have no competing interest.

## Authors’ contributions

SM performed the molecular analysis and wrote the manuscript; LM and BG supervised the study and critically revised the manuscript; EV, ED and RR performed the clinical characterization of patient and they are taking care of her; DT conceived the study, supervised it and wrote the manuscript. All authors read and approved the final manuscript.

## Pre-publication history

The pre-publication history for this paper can be accessed here:

http://www.biomedcentral.com/1471-2350/15/32/prepub
